# Coping with an antagonistic climate: Researchers' perspectives

**DOI:** 10.1007/s13280-025-02203-y

**Published:** 2025-06-14

**Authors:** Laila Mendy, Mikael Karlsson

**Affiliations:** Uppsala University, Geocentrum, Villavägen 16, 75236 Uppsala, Sweden

**Keywords:** Climate denial, Climate scepticism, Disinformation, Distrust, Science communication, Trust

## Abstract

Swedish researchers face an antagonistic climate of denial, scepticism, and distrust. Despite Sweden’s reputation as a climate forerunner, our interviews with 30 researchers show problems for conducting climate research in the country. Researchers use diverse communication and public engagement strategies, but often risk increasing exposure to problematic criticism and damage to their reputations. Interviewees considered themselves largely trusted, but reported attacks towards themselves and their research results. Anticipated misuse and unfounded criticism of research showed to be a decision factor for publishing. Threats and harassment have led to researchers self-censoring, avoiding media, or disengaging from public engagement. The societal implementation of research is disrupted by claims against researchers’ trustworthiness. This article suggests that researchers should be provided training, funding, and time to manage public engagement. Research institutions should take seriously the risks to their staff. Researchers should consider how different communication choices may affect their perceived trustworthiness to key audiences.

**Supplementary Information:**

The online version contains supplementary material available at 10.1007/s13280-025-02203-y.

## Introduction

The concept “post-truth” describes the polarisation, populism, proliferation of disinformation and rejection of scientific facts in contemporary society (Lewandowsky et al. 2017; Harsin 2018) in an era marked by sustainability crises (Folke et al. 2021). This phenomenon challenges environmental research and affects a range of environmental issues (Andersson et al. 2022; Starke et al. 2022). It is widely accepted that the misrepresentation of climate science is a major concern, as it challenges the scientific community, sows distrust in climate information and obstructs or delays climate mitigation efforts (Karlsson and Gilek 2020; Ekberg et al. 2022; Franta 2022; Gundersen et al. 2022; Hochachka 2024). Denial, scepticism, and distrust towards environmental research arguably constitute a “dark age” for the research community (Gundersen et al. 2022). Yet, few peer-reviewed studies have empirically explored how this issue affects the climate research community itself.

While Samoilenko and Cook (2023, 2024) categorised and discussed responses to the forms of attacks on climate researchers on contrarian blogs, the impacts of these attacks on researchers were not elaborated upon. In a case study, Lewandowsky et al. (2015) tracked the ways that denialist agendas seeped into and altered climate science research and debate. In a working paper, Sharman (2015) concluded that controversies impacted scientific agency, predominately in terms of increased caution and disruption, and called for further studies. Similarly, a recent review on counteraction of climate denial concluded that more research is needed to improve the understanding of how researchers “may engage with climate misrepresentations” (Mendy et al. 2024). Considering the potential impact of the dark age on science, we find it pertinent to explore whether and how climate researchers experience hostility towards themselves and their work.

Against the background of these research gaps, this article explores an antagonistic climate towards researchers and how they cope with it. More specifically, based on semi-structured interviews with 30 researchers, we answer the following research questions:

1. How have researchers in the climate field experienced negative reception to their work or their professions?
2. How do researchers cope with such experiences and issues?

We study these questions in the case of Sweden, which, given its long track-record of climate research and as a climate forerunner (Karlsson 2021), makes a pertinent case. Although known as a high trust society (Rothstein and Holmberg 2022), Sweden is not immune to contemporary trends of denial and scepticism in media (Vowles and Hultman 2021), the public (Jylhä et al. 2020) and political parties (Vihma et al. 2021).

We next present theoretical points of departure and the material and methods used. The results section elaborates on the tendencies discovered in the interviews and is followed by an analytical discussion. We finish with a set of conclusions relating the findings to the research context within Sweden.

### An antagonistic climate of denial, scepticism, and distrust

The literature on climate denial and climate scepticism is vast and includes different concepts, classifications and issues (Edvardsson Björnberg et al. 2017). Conceptually, some consider “denial” to be an appropriate term (Mendy et al. 2024), whereas others argue for using “scepticism” (Van Rensburg 2015). In the former group, Cohen (2001) differentiates between literal, interpretative and implicatory denial, which rejects, reinterprets and opposes the implications of facts, respectively. Rahmstorf (2004) further unpacks literal denial into rejection of trends, anthropogenic attribution, and impacts of climate change. In the latter group, Capstick and Pidgeon (2014) distinguish between the main types of epistemic scepticism towards climate science and response scepticism towards climate mitigation, whereas Van Rensburg (2015) and Jett et al. (2024) refer to evidence, process, and response scepticism. Additional distinctions can be found in the literature (Edvardsson Björnberg et al. 2017), but our interest in this article is neither to define nor take a stance on the specific characterisations; we instead consider them all to constitute an antagonistic climate that affects climate researchers.

The literature in the field evidently shows that climate researchers may face suspicion for a variety of reasons, whether for the evidence they produce, for the scientific processes they conduct or for their recommendations. This suspicion can moreover specifically target the reliability and trustworthiness of climate researchers (Coan et al. 2021; Cologna et al. 2024). While there is a general high and resilient trust in the scientific community (Cologna et al. 2024), individual researchers can be vulnerable to attacks that undermine them and their work. As researcher’s trustworthiness connects to their perceived benevolence, integrity, competence, and openness (Besley et al. 2021), climate deniers often apply a so-called “Serengeti strategy” of targeting and intimidating the individual researcher rather than the herd of the scientific community since that can be more effective than debating the findings from an entire scientific field (Mann 2015). For instance, Rojas et al. (2024) conclude that over half of contrarian climate claims on (then called) Twitter involve attacks on climate actors, including scientists. A study exploring contrarian blogs identifies five forms of ad hominem attacks against researchers: bias, moral, circumstantial, guilt by association, and competence (Samoilenko and Cook 2023). This suggests that a researcher’s perceived integrity and benevolence, which connect to bias or morality (Hendriks et al. 2015; Besley et al. 2021), are particularly vulnerable to ad hominem attacks. In the same vein, conspiracy theories about climate change researchers being personally motivated is also a belief held by sceptics (Lewandowsky 2014). Furthermore, while the “value-free ideal” of science demarcates it from politics (Gundersen et al. 2022), trends of co-production and participatory practices suggest that these boundaries are increasingly being blurred (Cologna et al. 2024), potentially providing fuel to accusations of researchers’ political bias.

The described antagonistic climate is largely due to actions by vested-interest actors in the form of disinformation (Cook et al. 2017; Lewandowsky 2021; Supran et al. 2023) often disseminated through social media (Coan et al. 2021; Rojas et al. 2024) and problematic new media practices (Edvardsson Björnberg et al. 2017). Media practices of cherry-picking or misbalancing debates are known forms of disinformation (Cook 2017). The problem may also be framed as a conflict between groups, connected to certain attitude roots and demographic factors, which indicate who is more likely to believe disinformation, reject climate change science, and delay mitigation (Norgaard 2006; McCright and Dunlap 2011; Bliuc et al. 2015; Hornsey and Fielding 2017; Huber et al. 2021).

In Sweden, climate denial and scepticism—typically expressed by between 6% (Oscarsson et al. 2021) and 10% of the population (Poortinga et al. 2019)—are connected to anti-feminism (Jylhä et al. 2020) and social-dominance personality (Jylhä and Akrami 2015). Far-right Swedish news sources are particularly antagonistic about the climate issue (Vowles and Hultman 2021), yet mainstream media practices could be considered problematic (Montpetit and Öberg 2021). Researchers regularly appear in media in Sweden, partly due to the “third mission”, a legal responsibility for publicly employed academics to disseminate research to society (SFS 1992:1434). Swedish academics are positively disposed towards public engagement, though often lack sufficient time for doing so (Bohlin 2019).

Though the literature offers suggestions about different strategies to counteract climate denial (Mendy et al. 2024), how researchers experience and cope with antagonism is less studied. Sharman (2015) identifies a spectrum of responses to scientific controversy, from engagement (such as rebuttal) to defensive avoidance (such as changing research topics). In response to ad hominem attacks, Samoilenko and Cook (2024) suggest investments in media literacy and science communication but it is not known how widespread such pre-emptive interventions are. Participatory processes can moreover mitigate distrust (Gundersen et al. 2022), partly because the researcher who willingly exposes themselves to antagonism is more trustworthy (Goodwin and Dahlstrom 2014), but it is not clear whether such insights are employed in practice by researchers.

To summarise, researcher’s may find themselves confronted with a variety of objections towards their work, their claims, or their role in society. Researchers’ trustworthiness can be attacked or undermined by different practices, potentially hindering information dissemination to stakeholders and the public. Our study contributes to this literature by offering novel empirical insights into how climate researchers experience and manage this antagonistic climate of denial, scepticism, and distrust towards themselves and their profession.

## Materials and methods

We rely upon interviews with 30 researchers working at Swedish research institutions in various scientific disciplines in relation to climate change and its socio-environmental implications. The interviewees in this study were systematically selected through first collating climate-related grants from two of the largest climate and sustainability research funding organisations in Sweden, after which the holders of the largest grants relating to climate change, mitigation, or adaptation were invited to interview. When unavailable, several researchers suggested a colleague. Of over 50 prospective interviewees 30 accepted (see Appendix S1). Participants remain anonymous in this paper.

Interviews were conducted between October 2023 and January 2024, semi-structured to garner understanding about how researchers perceive climate denial, to discuss their personal experiences, and to understand how they try to handle these issues (Appendix S2). The interviews lasted approximately 45 min and were conversational to encourage reflection, for instance, on the articulation of climate denial or alternative conceptualisations, with prompts to capture a breadth of experiences and perspectives (Adams 2015). The study was granted ethical approval (Swedish Ethical Review Authority, Dnr: 2023-03855-01). Transcribed interviews were inductively coded, descriptively and in vivo (Saldaña 2013), by both authors, striving for consensus coding. Codes were compiled, interactions categorised and inductively mapped in response to the research questions.

## Results

We found several tendencies but divergent perspectives were common, for example, on whether climate denial has increased or decreased in recent years, and the role of media. For instance, while every interviewee considered the denial of climate change to be an issue, for several this view only emerged once the definition was clarified to mean rejection of scientific evidence on the basis of non-scientific argumentation and not the welcomed critical inquiry of societal applications of science. Concepts such as “scepticism” and “distrust” were used interchangeably to describe interviewees’ observations or experiences. Furthermore, while most interviewees considered themselves largely trusted by the public and that they could engage with policymakers with relative ease, this high trust was not constant. Of the 30 interviewees, 21 had personally experienced or observed close colleagues in problematic encounters with publics or stakeholders. As the following subsections will show, interviewees seldom met criticism that addressed the content of their research but frequently experienced personal attacks in attempts to dismiss their results or scientific perspectives on an issue. Some suggested that while women and junior academics may be more vulnerable to attacks, with further qualifications and more public engagements academics increased their risk of exposure.

### Stakeholders and public engagement

Swedish researchers often present their work in public forums and seminars. In such contexts, interviewees experienced problematic objection to climate science, challenges to their work, and attacks on their credibility. Addressing objection takes time from valuable meeting spaces between researchers and stakeholders. Some interviewees said they had tried to shut down arguments, while others reported attempting to steer the conversation in more constructive directions. Trusting relationships between researchers and audiences were an important factor for constructive engagements.

One interviewee explained their encounter with right-wing politicians who attacked the legitimacy of climate researchers:

“We had a seminar about climate change, and there were politicians from the far-right…, and of course they went after all of us about legitimacy, and ‘you can’t say that’, and then ‘carbon dioxide is not the pollutant… blah, blah, blah.’ But I think we dealt with that quite nicely… not by saying ‘you're wrong’, because that creates a conflict… So, I said, ‘you might be right, that it's no problem. But you don't know that. You could be wrong. I could be wrong. In my world, we do something sensibly to deal with climate change, not by bringing us back to the Middle Ages, but to reduce emissions in a sensible way. And if I'm wrong, what will happen is that we have no carbon dioxide emissions, and the climate is fine. If you're wrong, the climate will be a disaster, and society will collapse. Do you want that?’ Sort of ‘No, I don't want that.’ No, but let's make sensible things, and we could agree.”

The interviewee encountered many forms of antagonism in this instance, yet chose to address the objections by constructively steering the conversation in order to maintain a positive relationship. However, this is not always possible, according to the interviewees, since some audiences may wish to disrupt the dissemination of research.

In the field of sustainable food systems, several interviewees recounted negative experiences with the farming community. One interviewee was accused of causing anaemia in young women by advocating for reduced meat consumption, an accusation met with standing ovation from the audience. After such accusations, researchers may find a constructive conversation unviable. Over time though, one of the interviewees had familiarised themselves with their farmer audiences and, when moderating questions, would avoid selecting those known to be disruptive. Another way mentioned to mitigate this hostility was to reframe messaging about reduced meat consumption as a “protein shift”, which according to one interviewee had been less contentious to stakeholders.

Interviewees indicated that the quality of the science produced did not necessarily enhance the uptake of information; it was the trusting relationships between partners that was seen to better facilitate the dissemination of scientific perspectives. To contrast two perspectives: one interviewee asserted that politicians generally are open towards researchers, especially when they have developed a relationship:

“Usually a policymaker wants to trust you, that you don't have a political agenda or trying to create problems for them, then they're fine. Do they think your research is trustworthy and willing to listen to it? I think that requires much more personal relationships. You have to build a personal relationship. It's not so much for them that you published in this journal or that journal or you use that method or some other method. I think it's more, you've been around for a long time, they've seen you for a long time, they trust you, that you know what you're doing and what you're saying is honest.”

Yet other interviewees explained how politicians had accused them of political bias in order to dismiss their work. In contrast with the above quotation, when meeting ministers about future climate scenarios, a politician posed a question about climate refugees that tied another interviewee to a left-wing political agenda. While frustrated, this interviewee had explained that this were outside their expertise.

We found that “relationship building” connects with increased positive reception to research. However, according to several interviewees, it requires that the researcher balances preserving relationships with critiquing problematic positions on the environment. One interviewee explained the risk that a researcher’s silence on an issue can be used to legitimise political positions. The same interviewee asserted that in their experience the reward of facilitating more opportunities for engagement was worth the commitment; audiences who are open to science from the start of a partnership often become more committed over time. Many interviewees, however, stated that they lack sufficient time for building relationships with politicians, stakeholders, and the public.

In summary, interviewees have met a range of objections to research in public contexts, which often takes time to address and can mean facing uncomfortable accusations. In some cases, researchers might mitigate negative reception through message framing or constructive dialogue. Such choices connect to decisions about upholding scientific integrity and which relationships to pursue. These challenges might affect the role of the researcher in society, particularly in terms of credibility and how much time they need for the “third mission”.

### Media appearance

Many interviewees discussed media appearances, including interviews or as opponents to Swedish politicians’ environmental proposals, which led to problematic experiences. Misrepresentation of research was common. Direct impacts included harassment from media during scientific controversies. Indirect impacts included personal attacks from the public.

Several interviewees explained how their research or statements had been mispresented in the media. One interviewee explained that following an interview, a media outlet had re-used their quotations in different contexts. Another interviewee found that their messages were improperly simplified and subsequently weaponised by politicians against an environmental organisation. Several interviewees explained that to avoid misrepresentation of their statements or science they were now cautious with the media.

Avoiding media did not guarantee a controversy-free research process, however. One interviewee explained how an influential right-wing personality wrote an op-ed that misrepresented their work by claiming that taxpayers had funded research about an unpopular social movement. The interviewee did not correct the misinformation but were dismayed at how widely the inaccuracy was accepted. As their colleagues had had similar experiences, resulting in “shit loads of bad comments”, the interviewee articulated a trend:

“Someone takes out like a chunk, like one of these like ‘Oh, this is a sexy message’, captures lots of readers and then this kind of gets spread but it loses the context.”

Researchers were also affected by controversies disconnected from them or their work. One interviewee recounted how a colleague got “thrashed” during a controversy between two media outlets. One of the outlets had misrepresented a scientific issue, which was criticised by another outlet:

“and they start calling him, and they're bringing in other experts that then criticise what he was talking about. It got so ugly."

Such instances demonstrate how media practices can impact researchers negatively. Interviewees explained that entertaining storytelling by antagonists may hinder the accurate dissemination of science, undermine the credibility of researchers, and weaponize science for political debates.

It was a common experience to receive upsetting emails or phone calls from members of the public after having appeared in broadcast media. An interviewee explained:

“Many times, it's also just an email saying ‘you're uneducated’, ‘you don't know what you talk about’, ‘you know nothing about climate change’, ‘shut up’ or ‘go to hell’, kind of thing.”

This phenomenon was common in the interviews. Another interviewee explained that after colleagues’ media appearances they “get a lot of climate denial questions and also, well, complaints that what they are doing is wrong and stupid and they are all idiots and things like that”. These quotations illustrate various forms of ad hominem attacks and name-calling. Despite nonchalance towards the likelihood of such emails, these attacks were felt personally by interviewees. Anticipating such emails discouraged several from accepting television invitations. When interviewees were asked how they respond to offensive or threatening emails, several seemed troubled by the obligation—for state agencies in Sweden—to respond. One suggestion was made to distinguish between emails with questions that should be responded to, and those that only made comments, which can be ignored.

One particularly nefarious experience described demonstrates the need to consider the reputation of the media organisation and risks of engagement. Earlier in one interviewee’s career they were interviewed by a popular news outlet from abroad:

“They asked me questions… I responded. It wasn't live. They then used my answers to make it sound like I was responding to different questions… So, it sounded like I wasn't answering. I was sort of, you know, not being to the point. It was really quite evil in the way that they set me up, basically.”

The interviewee was staged to sound vague and aloof, leading them to feel “set-up”. The outcome of this interview was rude emails and threats to the lives of the interviewee’s (yet unborn) children. The news organisation was subsequently black-listed at the research institution.

Trends have changed over time. According to the interviewees, a media controversy is more tiresome in the age of social media. At the time of the news “set-up”, social media was not widespread. The interviewee was grateful for this: “Thank God it wasn't, you know, this was before the age of Twitter and all that.” While interviewees explained that media’s misbalancing of the climate debate by pitting a scientist against a denier had become less common (though one interviewee continues to receive such invitations), the issue of media misrepresentation can now metastasise on social media. Yet interviewees experienced a range of problems due to media appearance, including attacks that intimidated several to silence. The examples provided by interviewees demonstrate how a multitude of media practices can expose researchers to unpleasant experiences.

### Online communication

Social media, particularly Twitter/X, was mentioned throughout the interviews. One interviewee characterised social media as a “major threat to society” due to its manipulation by those who “deny science or misuse science or misinterpret science and for a certain purpose”. This perception was common. Interviewees considered that being on social media unavoidably entails encountering problematic forces, impeding their “third mission” duty to disseminate research. Many interviewees explained that they had, particularly since Elon Musk’s purchase of Twitter, decided to leave the platform due to the unmoderated aggression they faced as researchers.

Using social media to disseminate research was common, yet many described an environment hostile to research. Concerning the field of forestry, for example, one interviewee explained that some colleagues avoided social media for research dissemination:

"…the forestry debate on Twitter was just [sound of bomb going off] … it was actually to an extent that my colleagues decided ‘I don't want to say anything about forestry on twitter because you only get negative responses.’ I tried, anyhow, and then I just muted some of those people. That’s just to keep my blood pressure normal."

Several of the interviewed researchers had chosen to disengage from social media. Interviewees motivated their withdrawal in terms of stress reduction, the hostile online discourse, by explaining that they had “had enough of idiots telling me that I was an idiot”, or found that these communication tasks were not justified in relation to work time and effects on their home life.

Comments received online were of similar ilk to those received after media appearances. Much of it was directed towards the researcher, rather than their research. Researchers’ habits were used to undermine their integrity. An interviewee explained that “for a while we had a diesel car and somebody looked that up and posted it on Facebook… and here is his address". Threats were made to family members. The interviewee interested in forestry explained they became worried about risks to their children due to such threats online. Accusations of incompetence or dishonesty were also hurtful. One interviewee, after having published something on Twitter/X, had read comments “like laughing at us that we wouldn’t know what we do, or we calculated things wrong, which was actually unpleasant”. Another interviewee suggested that they feared attacks based on integrity more than personal threats: "I would say what is worse… is calling me dishonest or a liar”. Despite the common frustration or anger of facing threats and personal attacks, it was noteworthy that some researchers were particularly affected by accusations targeting their competence and qualification.

The emotional impacts of social media were common, both in anticipation of and after receiving comments. One explained:

“I mean, it does something to you, right? Emotionally. It's like, makes you angry. It makes you more determined, perhaps, makes you want to be even better at explaining why the science says what it says. But it also makes, it has made me understand that there's a whole bunch of people who are really not interested in what the science says, just out of there to either insult and threaten or play, you know, gotcha kind of games."

In our interviews, researchers chose to either emotionally harden or to disengage. A lack of emotional or professional training opportunity was found to be problematic. Both personal experiences and their general observations of antagonistic social media had led interviewees to abandon social media for professional purposes.

Reputations online and offline may be symbiotically constructed. Yet, interviewees diverged about when and how researchers should share their perspectives online. One interviewee advocated for maintaining distinctions between political commentary and disseminating research. They explained:

"But you almost always separate between your research role and when you talk more about policy and what you think about policy. So, I’ve avoided social media not to become too political and still be able to be more of a researcher."

For them, an apolitical reputation online was important for protecting their relationships with decision-makers offline. A few interviewees mentioned this distinction. Several interviewees’ colleagues had discouraged them from speaking out on issues considered outside of their field. Those who had been critical to policy, indeed, found that used against them. One interviewee’s advocacy for stricter climate action was referred to in harsh attacks online. In this case, though, the emotional hardening of the researcher preceded attacks online; their “devastation” for climate mitigation was a driver of their communication.

Despite the value for science communication, social media exposed researchers to hostility. “Gotcha” games used personal lives, political statements, and researchers’ actions to undermine their scientific reputations. Researchers were angry, worried, stressed and found their home life impacted. Despite research institutions encouraging using social media, with the academic tendency to overwork interviewees felt there was weak justification to prioritise social media usage in such antagonistic settings.

### Conflict and dismissal

A common perception was that the position of a trusted scientist to whom politicians listen has increasingly been replaced by dismissal of science as irrelevant, particularly in socio-political conflicts. Some described this as a distinct form of objection to scientific information, which thus necessitated novel arenas for communication and public engagement. Several interviewees had chosen to write debate articles to mobilise action. Other interviewees, however, said that such articles undermine scientific authority and further degrade the trusted position of science in society.

One argument made against the debate format was the risk of reinforcing conflict between science and other societal perspectives. Interviewees explained that the result of such conflicts may be stakeholders “blocking off” from researchers who advocate for the truth against those who are “severely misled”. Indeed, for one interviewee op-eds authored by many researchers could worsen an already precarious situation for politically charged scientific fields by abusing public trust in research. On mass signatories, they said:

"They use their research position, I think, a bit too much. They speak about everything and just because they are a researcher they think they have the truth."

Several interviewees asserted restricting such activities to one’s expertise, especially in the current political context of Sweden, where a delegitimised researcher’s position could increase the likelihood of “politically governed” funding.

Those advocating such practices, however, were satisfied to see issues being discussed in society by maintaining scientific relevance in public debates. Beyond op-eds and debate articles, interviewees also mentioned researchers appearing on television to criticise or counter political positions. Some saw this as researchers being positioned against other trusted institutions, which may reinforce conflicts between science and other validity claims. Where interviewees had participated in such activities they were mindful of the limitations of the format.

### Research process

In our interviews, we found three ways that the research process had been disrupted: counter-environmental movements during the research process; the anticipation of backlash delaying publication; and outrage following publication that unsuccessfully attempted to force retraction.

There were examples of Swedish counter-environmental movements that targeted researchers. In order to not betray the identities of research participants we keep these moments vague. One interviewee’s colleague had received hundreds of phone-calls over the course of a year due to the publication of a report about misinformation and counter-environmental movements. Eventually the interviewee was also contacted, yet their attempts to rebut the raised concerns failed. The challenge was that the individual had an irreconcilable understanding of the issue.

Another explained that their participatory research process was disrupted when a political actor leaked information online:

"So, someone from the dialogue, among the dialogue participants had leaked the documents to this web editor, who had written an article about, and obviously denied some of the proposals that were made in the report, and raised questions… on legitimacy questions around the dialogue and so on."

However, the same interviewee also offered an alternative insight to disruption:

"I think that science should be happy about being denied. Because when we are denied, that means that we are relevant, that we are important, that we develop research about important things."

While interviewees often diverged in their views on the responsibilities of researchers to handle such issues, they converged around the importance of doing research about challenging and important issues. Disruption to research activities had not deterred them from pursuing their work. For several, controversies were rather an inspiration to pursue scientific research.

Two interviewees explained how potential problematic responses to specific research results were a contributing factor in decisions about publishing. We should note, however, that in both instances the hesitation was compounded by methodological flaws. One interviewee explained that they inappropriately used a method in a novel context and that the results suggested that the condition of an ecosystem was healthier than the scientific consensus. Anticipating that politicians would use the results to advocate for unsustainable extraction of natural resources, the interviewee ultimately decided to not publish results. Considering that their conclusions were based upon a flawed application of a method, the interviewee asserted that forgoing publication was appropriate. Concerns of political misuse of interviewees’ research were also shared by others. In a second example, a statistical miscalculation led to the research group panicking about stakeholder reception. According to the interviewee concerned, their team felt that “this will create so much hate, this would create such a horrible discussion that we, we can't.” Upon investigation, the error was corrected and results found to be reasonable. What this suggests is that researchers’ concerns about negative public reception or problematic political usage of science may influence publication decisions.

Scientific results that contradict normative understanding of an issue can lead to aggressive campaigns to force retraction. Antagonistic responses to scientific communication rarely related to specific results or conclusions, yet for one interviewee their specific results caused such international backlash that non-governmental organisations attempted to force retraction. The conclusions made were used to form conspiracy theories about extremist left-wing population control. While the university did not encourage retraction, the pressure continued for months. Media sustained the controversy’s impact on the interviewee during time away from work when, for example, they were contacted for comments on the university’s statement that advocated for academic freedom. Other interviewees referred to this case as an example of weak institutional support: one suggested that the university “threw [the researcher] under the bus”. While this case did not result in retraction, we consider it an example of a potential pressure point that signals how researchers may be silenced by their critics.

We found that an antagonistic climate towards research does not signify the issue uniquely as denialists versus scientists. Indeed, several interviewees explained that they had been called climate deniers due to finding results that contradicted mainstream understanding of an issue. One explained how upon publishing results “I got a letter from a [high official]: well, the conclusion you draw in your work is saying that there were no prison camps in Second World War Germany". Other interviewees linked similar accusations to political polarisation about climate mitigation. Where environmental activists were upset with research results they also had attacked interviewees, for instance, through the accusation of climate denial. In contrast to other findings, however, we found that these instances pertained to specific results rather than attempts to discredit a researcher’s influence.

#### Personal encounters

Most of the interviewees could share personal experiences of meeting someone who did not believe in climate change, some of whom were family members, parents of their children’s classmates, as well as hired workers. Meeting sceptical members of the public in personal networks could be upsetting but otherwise did not have wider impacts. Interviewees provided examples of when they had attempted to counteract such beliefs, though without successfully changing anyone’s mind about climate change. Many interviewees also avoided the discussion in order to maintain familial relationships.

Among the specific beliefs that surfaced in these contexts were doubts of the anthropogenic causation of climate change and beliefs that researchers fabricate problems for funding opportunities. Attempts to debunk these perspectives included explanations of the science of climate change and advocating that fellow scientists and their results were trustworthy. Often, particularly when related, the sceptical individual dismissed arguments about researchers’ trustworthiness with “well, I don’t mean you”. None of the interviewees could point to an occasion of having successfully changed someone’s opinion, despite constructive conversations. For the sake of relationships many instead avoided the topic of climate change.

## Discussion

We have found that when researchers disseminate their work, they may face ad hominem attacks, threats, and harassment. They can be accused of political bias or of wishing harm on society. Their work or statements can be misrepresented, their personal lives judged, and they and their family members threatened. These encounters can disrupt research and outreach efforts, demand considerable time, and are emotionally challenging. Researchers try to adapt to this antagonistic climate by constructively countering hostility, adopting new forms of communication, or disengaging from such settings. Despite variances in context, scientific field, and individual interpretation, several patterns have emerged, as summarised in Table 1. Column 1 lists the ways that researchers may interact with non-scientific audiences and be exposed to antagonism. Column 2 describes the impacts of these encounters. Column 3 presents different responses and strategies to adapt to or mitigate these challenges. Column 4 is further discussed below.

**Table 1:** Researchers’ experiences of antagonism and their coping strategies

How have researchers experienced negative reception to their work or their professions?		How do researchers cope with such experiences and issues?	
Form of interaction	Direct impact	Adaptation	Outcome and implication
Stakeholders and public engagement	Time wasted Attacks on credibility	Avoid conflict Select allies Balance critique	Relationship building Time cost Role of researcher
Media appearance	Misrepresentation Threats Harassment	Blacklisting outlets Collegial support Caution	Media training University support Staff training
Online communication	Threats Attacks	Self-censorship Emotional hardening	University support Role of the researcher
Conflict and dismissal	Science undermined	Debates and op-eds Communication change	Role of the researcher Relationship building
Research process and publication	Harassment Disruption	University response Publication change	University response Research process
Personal encounters	Emotional upset	Counteraction Relationship building	Role of the researcher Relationship building

The third mission in Sweden is an important responsibility of the researcher. However, our study demonstrates how antagonistic climates disrupt this task and takes time from already burdened researchers. Researchers may no longer rely upon linear science-to-policy channels (Karlsson and Gilek 2021). For researchers working with controversial environmental questions like climate change, different choices influence how various publics perceive their trustworthiness. There are few alternatives for reaching individuals predisposed to distrust researchers.

When understood as an inter-group conflict (Bliuc et al. 2015), the researcher stands on one side trying to persuade the antagonist on the other. From the interviewees’ perspectives and experiences, our findings suggest that researchers engage in a power struggle, seeking to assert their epistemic authority against forces keen to silence them. While scientists often need to uphold scientific integrity, positioning themselves as advocating the truth against those who are misled may only reinforce inter-group boundaries and impede constructive discussion. Some researchers indeed use science communication strategies to avoid triggering such conflict (Mendy et al. 2024), but these strategies often come with costs. Message framing, for instance, requires time and training for the researcher to identify and appeal to the underlying values of their audiences (Hornsey and Fielding 2017).

Many incidents described in this study pertained to characteristics of a researcher’s individual personality, indicating that antagonistic encounters can be understood as matters of distrust (Cologna et al. 2024). Researchers could, therefore, consider why they are less trusted by certain stakeholders and groups and try to find ways to address that, rather than the direct argumentation they are presented with. This consideration approaches the third mission task as less a matter of disseminating science, and more about building relationships between researchers and their publics.

It is up to the researcher, however, to determine to what extent they should disclose criticisms, and when to be quiet for the sake of improving dialogue. A researcher can enhance their trustworthiness by listening to concerns during engagements (Goodwin and Dahlstrom 2014). However, the public is not a monolith (Cologna et al. 2024) and different stakeholders have different underlying judgements of researchers and their actions. In contexts where researchers are generally trusted by their publics, as several interviewees asserted, pursuing relationships may be worthwhile to promote the uptake of scientific perspectives. However, where researchers are accused de facto of being biased or immoral, such pursuits may be impossible. In such instances, rather than appealing to antagonists (inadvertently legitimising criticisms), researchers can demonstrate scientific integrity to others present by not only providing knowledge, but also by acknowledging, for instance, limits to their knowledge or emphasising that they comment only on matters within their expertise. When political agendas are evident, researchers should be aware that their silence may also legitimise political positions.

When it comes to social media, despite the value for science communication, it is not a universally constructive enterprise. Researchers may find that online “gotcha” games can undermine their scientific reputation, echoing forms of ad hominem attacks found online (Samoilenko and Cook 2023). These incidences are stressful and impact the home life of the researcher. Research institutions that encourage social media use should increase training opportunities and provide support for potential problems. Researchers should consider these tasks in relation to other work activities and work–life balance decisions.

Environmental research is not conducted in a vacuum; external actors are vested in the processes and results of publication and not just in undermining the individual researcher. Battles between extreme political poles, including parties, media, and environmental and counter-environmental movements, have impacted how researchers can contribute to “informed, fact-based public debate” (Vihma et al. 2021). These battles have exacerbated the challenge of communicating scientific research and influenced research decisions. How researchers relate to these actors and groups at all stages of the research process is an important issue for the production of scientific knowledge. The media and research institutions play significant roles during times of controversy and may worsen already difficult situations. Yet, as researchers in the present study had varying levels of support from their communications departments, we consider that institutional assistance in these matters is sporadic in Sweden. Since research institutions often encourage media engagement, media training could help researchers understand the reputation of different outlets, how their data may be used, and how to cope with controversies or negative public reception.

## Conclusions

Our study shows that scientific agency has indeed been impacted by an antagonistic climate towards researchers in Sweden. We demonstrate how researchers are exposed in different communication contexts, leading to a variety of impacts on researchers. Our findings offer empirical support to the idea that this phenomenon has impacted the production of research, from anticipation of problematic reception to post-publication. Research institutions should take note of risks to their staff in these contexts. When institutions encourage their staff to expose themselves to these risks, which we consider important, providing institutional resources and training to handle such encounters is essential.

Our first research question regarded how researchers have experienced negative reception to their work or their professions. We found that in an antagonistic climate, researchers have experienced hostility from a wide number of non-academic actors, in as many contexts as researchers choose to disseminate their perspectives, and for a number of reasons. The impacts were both personal and professional. Attacks to their reputation, particularly accusations of bias and immorality, for example, were found to disrupt important communication processes. The prevalence of such reflects the nature of ad hominem attacks online (Samoilenko and Cook 2023). These drained time and energy and limited resources for academics already lacking such for communication (Bohlin 2019). Claims of incompetence or dishonesty, or threats to themselves and loved ones were stressful and upsetting.

Regarding the second question, about how researchers cope with such issues, we found that researchers adopt both preventative and adaptive strategies to reduce negative reception. This includes changed communication practices, such as avoiding audiences, employing message framing, or forgoing criticism or debate for maintaining relationships. Others have asserted the value of taking debate. While advocacy for climate action, not found to necessarily affect epistemic trust (Cologna et al. 2024), can indeed be used in accusations of bias, researchers can meet such accusations without any advocacy. Improved participation was believed to become more productive over time, arguably an antidote to distrust (Gundersen et al. 2022). Research has also been impacted by both the anticipation of and negative reception to scientific results.

We have found that researchers must navigate complex contexts of division where they may be unwittingly drawn into controversy, echoing Sharman (2015). There are a number of decisions to take to best engage while protecting reputations or enhancing potential future fruitful engagement. Researchers aspire to cultivate durable relationships but struggle with minimal support from their institutions. They also may disagree about where their voices are inappropriate and what different activities, such as debate articles to hold politicians to account, may do to their community’s reputation.

Our findings uniquely focus on the experiences and impacts of an antagonistic climate on researchers and their professional activities, based upon a Swedish interview study. We have found widespread and diverse impacts, but considering the prevalence of climate denial in many countries (Edvardsson Björnberg et al. 2017) we encourage studies from international contexts. Furthermore, our findings suggest the value of more research exploring how researchers may be informed or affected by the perceptions of their audiences, the anticipation of critical attitudes and how this may influence researchers’ research practices.

This issue is complex and, according to Gundersen et al. (2022), does not come with simple solutions: researchers have very divergent understandings about the issue and their responsibilities to navigate it. Our study sheds light on some of the different contexts where important decisions can be made, where activities are particularly under critical scrutiny, and the choices researchers can make.

## Supplementary Information

Below is the link to the electronic supplementary material.

Supplementary file1 (PDF 733 KB)

## Data Availability

The participants of this study did not give written consent for their data to be shared publicly, so due to the sensitive nature of the research data is not available.
