# Supplementary material for: Coping with an antagonistic climate: Researchers' perspectives
Source: Ambio. 2025 Jun 14;54(12):2190–201. doi: 10.1007/s13280-025-02203-y (PMC12569219; doi:10.1007/s13280-025-02203-y)
Supplement: Supplementary file 1 — Supplementary file1 (PDF 733 KB) [file 13280_2025_2203_MOESM1_ESM.pdf]

SUPPLEMENTARY INFORMATION

Supplementary Information
This supplementary information has not been peer reviewed.

Journal Name: ***Ambio***

Title: **Coping with an Antagonistic Climate: Researchers' perspectives**

Authors: **Laila Mendy and Mikael Karlsson**

Corresponding Author:

Laila Mendy, department of Earth Sciences, Uppsala University

Address: Geocentrum, Villavägen 16, Uppsala, 75236

Email: laila.mendy@geo.uu.se

Appendix S1. Selection Protocol

We were interested in exploring the ways researchers in Sweden – in natural, social and interdisciplinary sciences engaged with climate change and mitigation in a broad sense – perceive, experience and cope with climate denial, scepticism and distrust. We wanted to identify researchers in a broad range of disciplines, active in larger climate-related projects ongoing at the time of the study.

We first selected a number of climate-related calls for research funding, issued by two major research funders in Sweden, and identified a list of the programs and projects that had been granted the highest levels of funding over the past five years (since 2018). We then picked over 50 researchers (from junior researchers to more senior, e.g. Professors and Associate Professors) from the programs and projects, ending up with diversity in terms of gender, age, discipline, and institution. These researchers were invited to the study and asked to share the invitation to their colleagues where they were unable or uninterested, which happened in a number of cases.

To ensure anonymity, we do not disclose the lists, nor the names of the interviewees, but they were active in one or several of the following disciplines and research areas: sustainability science and environmental science; political science and policy; sociology and philosophy; economics and environmental economics; health; children; engineering, tourism, communication; biology, ecology and biogeochemistry; transportation, food, soil and forests; climate mitigation and adaptation.

Appendix S2

Question Guideline

**Theme**	**Questions**	**Follow ups/ prompts**
Formalities	Can you provide your name, your role, and work place?	
	Can you explain a little about your research?	How do you work practically?
	How do you communicate or disseminate your results?	Prompt: media, social media, boards/ chairs, popular summary.
General Perspectives	Do you consider the denial or rejection of science to be a problem for society?	Why/ Can you elaborate?
	Can you explain how science is trusted in society? (What does it mean to trust science?)	What makes science/ the researcher trustworthy? /How does this form of trust relate to science denial?
	How might this impact or change the role of the researcher/ research in society? Can you exemplify?	
	Do you consider science denial to relate to the achievement of Sweden's environmental goals?	How?
Personal experiences	Is research from your field criticized from outside of academia, or denied completely? Can you provide examples?	What do you consider these objections to depend upon?
	Have you personally experienced such forms of critique, objections, or denial? In what instances?	Where did these objections come from, in which channels/ settings, and how were they expressed?
		How did you react to these objections? How did you experience them personally?
	Have you ever been threatened, harassed, or such (related to your research)?	Can you explain what happened?
	Have any of your colleagues experienced climate denial?	How do you observe the way they relate/ related to it?
	Do you have experience in actively countering science denial?	How did you it go? Can you explain when you might have succeeded – what did that look like?
	Has (potential) objection to your research ever impacted your research choices?	Can you explain when and why? Prompt: communication, framing, collaborators
Counteraction	What is the responsibility of the researcher in handling these issues?	What support or knowledge would you need to do this?
	How does your university or institution handle these issues?	
	Which other actors have a role or responsibility in these issues?	Prompt: media, politicians, business, funding, and other organisations
Closure	Is there anything more you would like to explain linked to and beyond these questions?	
